# Development and Validation of a High-Performance Liquid Chromatography Method to Quantify Marker Compounds in *Lysimachia vulgaris* var. *davurica* and Its Effects in Diarrhea-Predominant Irritable Bowel Syndrome

**DOI:** 10.3390/molecules29071489

**Published:** 2024-03-27

**Authors:** Hye-Youn Kim, Cho-Een Kim, Dool-Ri Oh, Yonguk Kim, Chul-Yung Choi, Jaeyong Kim

**Affiliations:** 1 Jeonnam Institute of Natural Resources Research (JINR), Jeonnam Bio Foundation, Jangheung-gun 59338, Republic of Korea; khyoun412@naver.com (H.-Y.K.); superman313@jbf.kr (C.-E.K.); pp0806@jbf.kr (D.-R.O.); kyu9801@jbf.kr (Y.K.); 2 BK21 FOUR Educational Research Group for Age-Associated Disorder Control Technology, Institute of Well-Aging Medicare, Department of Integrative Biological Sciences, Chosun University, Gwangju 61452, Republic of Korea; blockstar@chosun.ac.kr

**Keywords:** *Lysimachia vulgaris* var. *davurica*, irritable bowel syndrome, high-performance liquid chromatography, marker compounds, diarrhea, validation

## Abstract

Irritable bowel syndrome (IBS), a common gastrointestinal disorder worldwide, is characterized by chronic abdominal pain, bloating, and disordered defecation. IBS is associated with several factors, including visceral hypersensitivity, gut motility, and gut–brain interaction disorders. Because currently available pharmacological treatments cannot adequately improve symptoms and may cause adverse effects, the use of herbal therapies for managing IBS is increasing. *Lysimachia vulgaris* var. *davurica* (LV) is a medicinal plant used in traditional medicine to treat diarrhea. However, information on whether LV can effectively improve diarrhea-predominant IBS (IBS-D) remains limited. In this study, using an experimental mouse model of IBS-D, we elucidated the effects of the LV extract. The methanol extract of LV decreased fecal pellet output in the restraint stress- or 5-hydroxytryptamine (5-HT)-induced IBS mouse model and inhibited 5-HT-mediated [Ca^2+^]*_i_* increase in a dose-dependent manner. Furthermore, we developed and validated a high-performance liquid chromatography method using two marker compounds, namely, chlorogenic acid and rutin, for quality control analysis. Our study results suggest the feasibility of the methanol extract of LV for developing therapeutic agents to treat IBS-D by acting as a 5-HT_3_ receptor antagonist.

## 1. Introduction

Irritable bowel syndrome (IBS), a common functional gastrointestinal disorder, is characterized by chronic abdominal pain, distention, and disordered defecation (constipation and/or diarrhea) in the absence of a known organic pathology [1,2]. The prevalence of IBS is estimated to be 11.2% worldwide, with the highest prevalence in South America (17–21%) and the lowest in South Asia (7–9%) and Asia (10–20%) [3,4,5]. Although the etiology of IBS remains unclear, several factors, including genetics, visceral hypersensitivity, immune-mediated factors, gut motility, alterations in gut microbes, gut–brain interaction disorders, and psychosocial factors, may contribute to its symptom complex [6,7]. Based on the predominant stool patterns, IBS is divided into four subtypes: IBS with constipation (IBS-C), IBS with diarrhea (IBS-D), mixed IBS, and unsubtyped IBS. In general, IBS treatment is based on the predominant symptoms [8,9]. The treatment strategies for IBS-C are targeted toward relieving constipation. Linaclotide, plecanatide, guanylate cyclase-C receptor agonists, and lubiprostone, a type 2 chloride channel activator, are used as pharmacological agents. Furthermore, the treatment strategies for IBS-D are targeted toward relieving diarrhea. Eluxadoline, a μ and κ opioid receptor agonist and δ-receptor, alosetron, a selective 5-HT_3_ receptor antagonist, and rifaximin, a gut microbiota modulator, are used as pharmacological agents [9]. In addition to pharmacological therapy, IBS is primarily treated by modifying the diet, providing psychological assistance, and making lifestyle changes, including changes in dietary habits and physical activities [10].

At present, pharmacological treatments are only based on symptom reduction and carry the risk of side effects [11]. For example, constipation is the most frequent adverse event of both alosetron and ramosetron, which are 5-HT_3_ receptor antagonists for the treatment of IBS-D, and side effects such as headache, dry mouth, and dizziness have been reported [12,13]. Therefore, the use of complementary and alternative medicines to treat IBS, particularly herbal therapies, is increasing [14]. In vitro and preclinical studies as well as clinical trials have revealed the effects and acceptable safety of various plant sources in improving IBS. Meta-analyses have shown that *Aloe vera*, *Mentha piperita*, *Cynara scolymus*, and *Perilla frutescens* significantly improved symptoms compared to placebo in patients with IBS and increased their quality of life scores. These herbs can be used alone or in combination with conventional medications to decrease drug side effects and overcome drug resistance [15].

*Lysimachia vulgaris* var. *davurica* (Ledeb.) R.Knuth (LV) is a rhizomatous perennial plant that grows in moist mountainous grasslands and near streams and waterways throughout Europe and Asia [16,17]. LV has been used to treat ulcers, fever, inflammation, and diarrhea in traditional medicine. Recently, studies have revealed that LV extracts exhibit antioxidant, antibacterial, antitumor, and wound-healing activities [18,19,20]. Furthermore, young LV leaves are edible and registered as a food ingredient in East Asia [21]. In terms of its chemical composition, LV contains phenolic acids, including chlorogenic acid and *p*-coumaric acid, and flavonoid glycosides, including hyperoside, isoquercitrin, and rutoside [22]. Studies suggest that polyphenols are potential therapeutic phytomedications for preventing and improving IBS symptoms [15,23,24].

Consistency in chemical composition and biological activities is vital for safely and effectively using herbal drugs and natural resources. Marker compounds are chemically defined components present in herbal drugs that can be used for the quality control of herbs regardless of their therapeutic activity [25]. To ensure substance consistency, marker-based standardization of herbal medicines, which involves the identification of unique and primary plant components as markers and the development of analytical methods to monitor them, is becoming increasingly common [26]. As an astringent, LV is primarily used to treat gastrointestinal diseases such as diarrhea and dysentery; however, information on its effectiveness and underlying mechanisms is limited [27]. To the best of our knowledge, there have been no experiments measuring the improvement of diarrhea in a mouse model with increased gastrointestinal motility using LV extract, and no prior research has been conducted on the mechanism through which this effect might occur. This study hypothesized that LV could be utilized as a novel natural material with efficacy in improving diarrhea. To test this hypothesis, we elucidated the effects of the LV extract using a mouse model of IBS-D. We examined the effects of water and methanol extracts of LV on the fecal pellet output induced by restraint stress or 5-HT, which increases intestinal motility, and the inhibitory action of LV extract on 5-HT_3_ receptor-induced [Ca^2+^]*_i_* increase, which is associated with functional gastrointestinal diseases such as IBS. Furthermore, marker compounds were identified to standardize the LV extract using ultra-performance liquid chromatography (UPLC)–quadrupole time-of-flight mass spectrometry (Q-TOF MS), and a high-performance liquid chromatography (HPLC) method was developed and validated to simultaneously monitor and quantify these marker compounds.

## 2. Results and Discussion

### 2.1. Effect of the LV Extract on Fecal Pellet Output in Mice

First, we elucidated the effect of a single oral administration of the LV extract on restraint stress-induced fecal pellet output in mice. Two LV extracts were tested: water and methanol.

**Figure 1:**
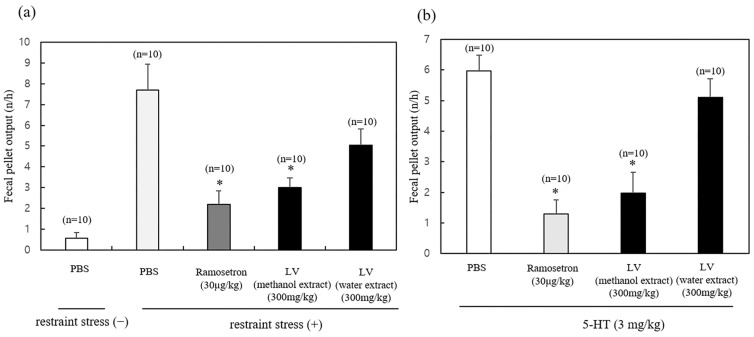
Effects of *Lysimachia vulgaris* var. *davurica* (LV) extract on restraint stress- or 5-hydroxytryptamine (5HT)-induced changes in fecal pellet output in mice. Mice were orally administered 300 mg/kg of the LV extracts (methanol and water). After 2 h, mice were exposed to (**a**) restraint stress or (**b**) 5-HT (3 mg/kg, I.P.). The number of fecal pellets excreted in 1 h was determined. Data are presented as mean ± standard deviation (*n* = 10). * *p* < 0.05 as compared with PBS.

In untreated mice (administered phosphate-buffered saline [PBS] vehicle only), restraint stress increased fecal pellet output per hour by more than five-fold compared with unrestrained mice (Figure 1a). Ramosetron (30 µg/kg, positive control, p.o.) significantly inhibited stress-induced fecal pellet output in mice. Furthermore, oral administration of the LV methanol extract (300 mg/kg) suppressed fecal pellet output better than the water extract (300 mg/kg).

Next, we determined the effect of the LV extracts on 5-hydroxytryptamine (5HT) (3 mg/kg)-induced fecal pellet output, which increases intestinal motility. Figure 1b demonstrates that oral administration of the LV methanol extract (300 mg/kg) suppressed 5HT-induced fecal pellet output.

IBS is a common gastrointestinal disorder worldwide. In particular, IBS-D is more prevalent in Western countries and Asia [28]. According to traditional medicine, LV has been used to treat diarrhea since ancient times [29]. Other studies have used animal models of bowel dysfunction induced by restraint stress or stress-related drugs, including serotonin [29,30]. Our data suggest that the LV methanol extract can be used to suppress IBS-D. More in-depth research into the mechanism of its action still needs to be carried out.

### 2.2. Inhibitory Effects of the LV Methanol Extract on 5-HT_3_ Receptor-Induced [Ca^2+^]_i_ Increase

Among 5-HT receptors, the 5-HT_3_ receptor is the only ligand-gated ion channel that is expressed in various brain regions, including the hippocampus, cortex, and gastrointestinal tract [31]. The 5-HT_3_ receptor is highly permeable to extracellular Ca^2+^. When treated with an agonist (5-HT), it increases intracellular Ca^2+^ influx [32].

**Figure 2:**
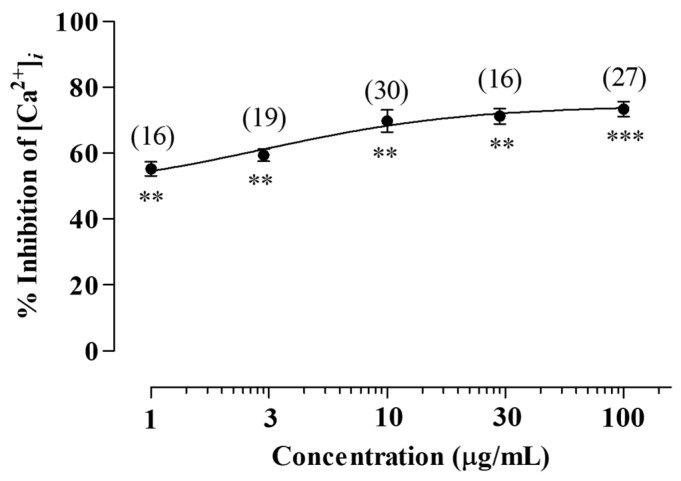
Effects of the LV extract on 5-HT_3_ receptor activity in human 5-HT_3_ receptor-transfected CHO-K1 cells. CHO-K1 cells were transfected with the human 5-HT_3_ receptor and loaded with fura-2-acetoxymethyl ester. Cells were pretreated with the LV methanol extract for 1 min and treated with 5-HT (100 µM). ** *p* < 0.01 and *** *p* < 0.001. 5-HT_3_, serotonin receptor subunit 3; CHO-K1, Chinese hamster ovary K1 cells; and 5-HT, serotonin.

In this study, the LV methanol extract exhibited the strongest inhibitory effect on 5-HT_3_ receptor-induced [Ca^2+^]*_i_* increase. At concentrations of 1, 3, 10, 30, and 100 µg/mL, the LV extract inhibited 5-HT-induced [Ca^2+^]*_i_* increase in a dose-dependent manner to 55.23% ± 2.12%, 59.40% ± 1.81%, 69.77% ± 3.31%, 71.22% ± 2.35%, and 73.39% ± 2.20%, respectively (Figure 2). Furthermore, the half-maximal inhibitory concentration was 3.40 ± 1.50 μg/mL. These results suggest that the LV methanol extract exerts an inhibitory effect on 5-HT_3_ receptor-induced [Ca^2+^]*_i_* increase, indicating 5-HT_3_ receptor antagonism.

5-HT_3_ receptor antagonists prevent antineoplastic chemotherapy-induced nausea and vomiting as well as neurodegenerative, inflammatory, and psychiatric diseases [33]. Furthermore, they can be used to treat IBS-D [34]. Clinical trials have revealed that alosetron and cilansetron, both 5-HT_3_ receptor antagonists, can significantly improve abnormal bowel function and relieve pain and discomfort in men and women with IBS-D [34,35]. Collectively, the findings suggest that the LV methanol extract is an effective therapeutic agent for IBS-D that acts via the 5-HT_3_ receptor antagonistic mechanism.

### 2.3. Identification of Marker Compounds in the LV Methanol Extract

**Figure 3:**
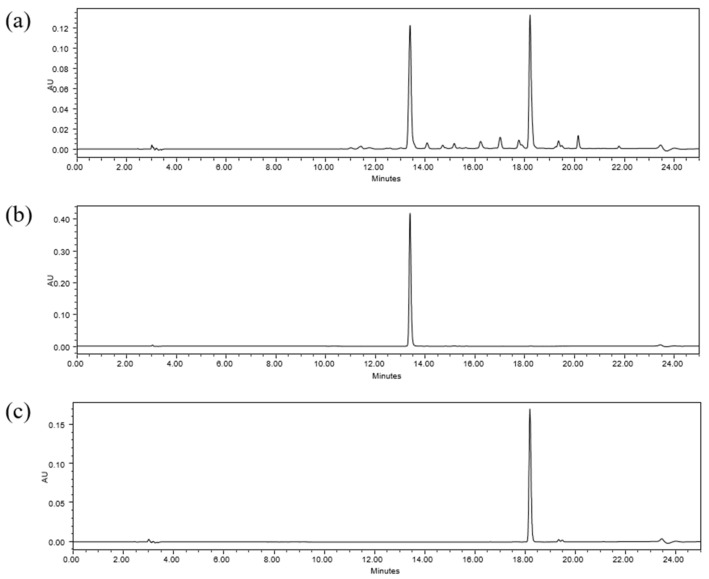
Chromatograms at 324 nm of (**a**) LV methanol extract, (**b**) chlorogenic acid, and (**c**) rutin.

**Figure 4:**
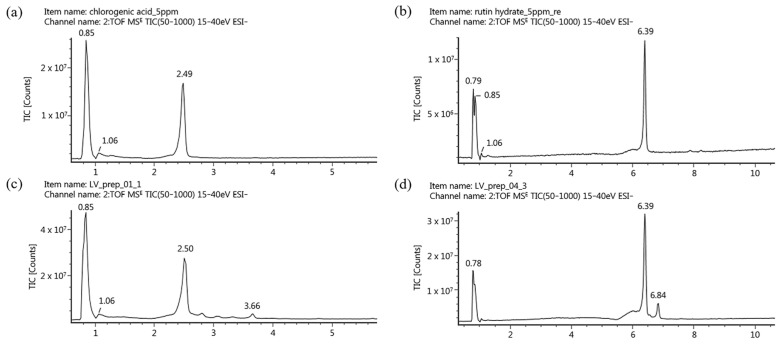
Total ion chromatograms in negative ion mode (from UPLC–Q-TOF–MS) of the standard solutions of (**a**) chlorogenic acid and (**b**) rutin as well as (**c**) fraction 1 and (**d**) fraction 2 from the LV extract. Fractions 1 and 2 were obtained from the crude extract based on the major peaks identified via HPLC–PDA.

**Figure 5:**
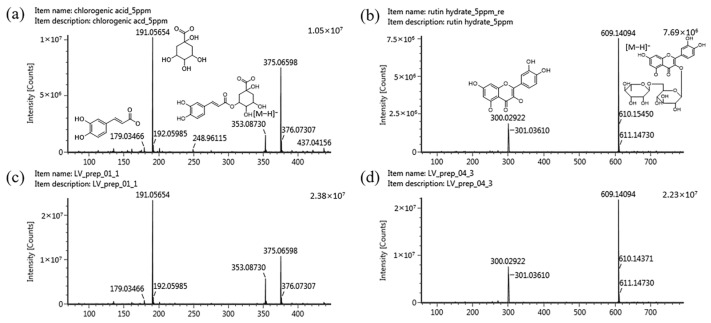
MS/MS spectra of the standard solution of (**a**) chlorogenic acid and (**b**) rutin as well as (**c**) fraction 1 and (**d**) fraction 2 from the LV extract. MS analysis was performed on a Q-TOF–MS/MS system combined with an electrospray ionization source in negative ion mode.

In order to use natural products as raw materials, functional foods, and cosmetics, quantifying their marker compounds is vital to ensuring quality, efficacy, and reproducibility. Marker compounds vary depending on geographical, seasonal, and genetic factors [36]. In the present study, we selected marker compounds for the standardization of LV representing the raw material based on representativeness, stability, and ease of analysis, prioritizing those with the highest content in the extract using HPLC. HPLC–photodiode array (PDA) detection was used to monitor and isolate the primary components in the LV methanol extract. Through UPLC–PDA–Q-TOF MS analysis, two major marker compounds were identified in the isolated fractions. Figure 3a illustrates the representative chromatogram of the LV methanol extract; two primary peaks of abundance were observed at 13.3 and 18.2 min. Two fractions were obtained by manually collecting these main peaks at the time of their elution. To identify the primary peaks in the HPLC profile of LV, two approaches were applied in LC-MS/MS. First, using the UNIFI screening platform, high-resolution *m*/*z* and mass fragment ions (MS/MS spectra) were analyzed against databases, followed by confirmation using standard compounds. As a result, chlorogenic acid and rutin were identified as the marker compounds of LV. The retention times of the two standard compounds matched those of the peaks in the isolated fractions (Figure 4). The corresponding mass spectra exhibited *m*/*z* values for the precursor ions at 353.0873 [M − H]^−^ and 609.1409 [M − H]^−^, respectively, indicating deprotonated chlorogenic acid and rutin. The most significant fragment ions identified were *m*/*z* 191.0565 and 179.0346 in the MS2 spectrum of chlorogenic acid and *m*/*z* 301.0361 and 300.0292 in the MS2 spectrum of rutin. Figure 5 presents the MS2 spectra and proposed structures of the predominant fragment ions. Previous studies have revealed and compared the polyphenolic compositions of *Lysimachia* species, including *Lysimachia nummularia* L., *Lysimachia vulgaris* L., and *Lysimachia punctata* L., using the HPLC–diode array detection and HPLC–MS methods [19,22,37,38]. Polyphenolic compounds such as chlorogenic acid, p-coumaric acid, hyperoside, isoquercitrin, quercitrin, and luteolin were identified in LV, and chlorogenic acid was only detected in the LV extract; therefore, it can serve as a marker for detecting the adulteration of these species [22,38]. A previous study revealed that rutin is the dominant compound in the methanol extracts of field-grown and in vitro-grown LV, which is consistent with our present findings [19]. Furthermore, a recent study has suggested that chlorogenic acid inhibits the NF-κB pathway, which is involved in intestinal epithelial barrier dysfunction, by downregulating CD14 and p65 [39]. The intestinal barrier plays an essential role in intestinal homeostasis; its disruption may lead to inflammatory bowel disease (IBD) owing to excessive immune responses against the gut microbiota [40]. In addition, chlorogenic acid can alleviate post-infectious IBS symptoms in rats by regulating the gut microbiota and its metabolites [41]. Rutin is a polyphenolic compound with potent antioxidant and anti-inflammatory effects [42]. Dietary rutin can ameliorate dextran sulfate sodium-induced colitis in mice by decreasing the production of proinflammatory cytokines, including interleukin (IL)-1β, IL-6, granulocyte–macrophage colony-stimulating factor, and inducible nitric oxide synthase [43]. Furthermore, Kim et al. have reported that rutin can attenuate 2,4,6-trinitrobenzene sulfonic acid-induced colitis in rats. They suggested that rutin delivers quercetin to the large intestine and that quercetin suppresses TNF-α-induced NF-κB activation, a major inflammatory pathway involved in IBD pathogenesis [44]. In the present study, chlorogenic acid and rutin, the marker compounds in the LV methanol extract, were identified using UPLC–PDA–Q-TOF MS. Previous studies have suggested that these compounds can ameliorate IBS. To standardize LV quality, we developed an HPLC–PDA method, a widely used general-purpose detection system. Method validation was performed using parameters such as specificity, linearity, limit of detection (LOD), limit of quantification (LOQ), precision, recovery, and solution stability according to ICH guidelines.

### 2.4. Validation of the HPLC Method

#### 2.4.1. Specificity

Specificity refers to the ability to evaluate whether the identification and quantification of the compound of interest are affected or not by the presence of other substances in the samples [45]. Blank solutions, LV extract samples, and standard solutions of chlorogenic acid and rutin were analyzed using the developed HPLC–PDA method. Figure 3 illustrates the chromatograms obtained for the samples and each standard. Chlorogenic acid and rutin were detected at approximately 13.35 and 18.19 min, respectively, in the samples and standard solutions. The retention times of the marker compounds in the sample were similar to those of the standard compounds. By comparing the chromatograms of the standard compounds and the LV extract, we confirmed that the peaks of chlorogenic acid and rutin were well separated and with no interference from other components of the extract, indicating the specificity of the developed HPLC method.

#### 2.4.2. Linearity, LOD, and LOQ

Linearity was simultaneously established using six concentrations ranging from 2.5 to 100 μg/mL for chlorogenic acid and rutin. Table 1 shows that the linear equations of the two marker compounds were y = 47,062x − 39,362 and y = 20,210x − 1351, with correlation coefficients (r^2^) of 0.99969 and 0.99999, respectively. An r^2^ value of >0.99 is generally considered adequate, and our results demonstrated a high linearity of ≥0.999 within the test range [46,47]. LOD and LOQ are the minimum concentrations of an analyte in a sample that can be reliably detected or quantified, respectively [45]. Using the developed method, the LOD and LOQ of chlorogenic acid were 0.39 and 1.17 μg/mL, respectively, allowing the quantification of 24.3 μg/mL in the sample. In contrast, the LOD and LOQ of rutin were 0.27 and 0.82 μg/mL, respectively, allowing the quantification of 54.9 μg/mL in the analyzed sample (Table 1).

#### 2.4.3. Intra- and Inter-Day Precision

Precision refers to the degree of similarity of the independent measurement results obtained through repeated experiments. Several factors, including sample preparation, injection reproducibility, chromatographic conditions, and analyst skills, can affect precision [48,49]. The intra- and inter-day precisions of chlorogenic acid and rutin were verified in at least three replicates using 5, 25, and 50 μg/mL concentrations in the linear calibration range. The intra- and inter-day precision in terms of relative standard deviation (RSD) were 0.07–0.39% and 0.15–0.32% for chlorogenic acid and 0.45–0.75% and 0.24–0.32% for rutin, respectively (Table 2). The recommended RSD to ensure the precision of the analytical method is generally <2%, and RSDs of 5–10% are generally acceptable at the LOQ [50]. In conclusion, the RSD values of chlorogenic acid and rutin were <2%, indicating the good precision of our developed method.

#### 2.4.4. Recovery

The accuracy of an analytical procedure describes the similarity between the experimental value and the actual amount of a substance in the matrix. It is evaluated using the mean percentage recovery by measuring a known amount of an analyte added to the sample [45]. In the present study, the sample extract was spiked with standards at three different concentrations (5, 25, and 50 μg/mL). The percentage recoveries were 104.47%, 101.65%, and 101.61% for chlorogenic acid and 103.53%, 100.15%, and 102.78% for rutin at the low, medium, and high concentrations, respectively, while the RSD values were <2% (Table 3). This accuracy is acceptable considering that the recovery values were 90–107% for our studied concentration levels based on AOAC recommendations [51]. Accordingly, these results confirm that the established method for chlorogenic acid and rutin exhibits good accuracy.

#### 2.4.5. Solution Stability

Solution stability helps to determine whether normal conditions, normal storage conditions, and special storage conditions, including refrigeration or protection from light, are needed [52]. In most HPLC analyses, the sample solution is typically injected overnight using an autosampler; therefore, if solution pretreatment and storage require more than 24 h, it is crucial to establish a period during which the test solution can be used stably. In the present study, standard and sample solutions were stored at room temperature and 0–4 °C and the percentage differences in the peak areas of chlorogenic acid and rutin were determined at different time intervals (12, 24, and 48 h). Table 4 summarizes the percentage differences in absolute values for chlorogenic acid under room temperature and 0–4 °C conditions from 12 to 48 h, which were 0.13–1.34% and 0.06–1.18%, respectively. The percentage differences in absolute values for rutin under room temperature and 0–4 °C conditions from 12 to 48 h were 0.08–0.59% and 0.02–0.52%, respectively. The percentage differences in the peak areas of chlorogenic acid and rutin in both the standard and sample solutions were <2%, suggesting that the prepared solutions were stable for up to 48 h at RT and 0–4 °C conditions.

#### 2.4.6. Quantification of the Marker Compounds in the LV Methanol Extract

The validated HPLC method was used to determine the concentrations of the two marker compounds, chlorogenic acid and rutin, in the LV methanol extract. The concentrations of chlorogenic acid and rutin in the LV methanol extract were 24.31 and 54.89 mg/g, respectively. The RSD values for chlorogenic acid and rutin were 0.95% and 0.69%, respectively (Table 5). Previous studies have revealed the concentrations of chlorogenic acid and rutin in the LV extract and fractions. The chlorogenic acid concentration was reported to be 31.6 mg/g in the silica gel fraction of *L. vulgaris* and 78.0 μg/g in the ethanol extract [22,38]. Furthermore, the rutin concentration in the methanol extract of field-grown LV from Turkey was 29.0 mg/g [19]. Polyphenols are molecules involved in stress protection in plants, and exhibit increased concentrations in plants exposed to stressors such as extreme temperatures, light, salt, drought, and floods [53]. Therefore, the different concentrations of these two compounds in LV in each study may be associated with their growth conditions in the natural environment.

In the present study, we developed and verified an analytical method to easily and rapidly quantify marker compounds in LV methanol extract using HPLC–PDA, a general-purpose detection technique. Our study results suggest that the developed analytical method is useful for setting quality control standards and monitoring the quality of LV raw materials for the use of natural resources in the pharmaceutical and nutraceutical industries.

## 3. Materials and Methods

### 3.1. Plant Material and Chemicals

The aerial parts of LV used in this study were collected from Jangheung-gun, Jeollanam-do, Republic of Korea (34°40′01.7″ N 126°56′44.2″ E) and deposited at the herbarium of the Jeonnam Institute of Natural Resources Research (JINR), (voucher specimen number JINR2108100001). They were authenticated by Dr. Kim (JINR, Jangheung-gun, Jeollanamdo, Republic of Korea). 5-HT was purchased from Nacalai Tesque (Kyoto, Japan). HPLC-grade methanol, acetonitrile, and water were procured from JT Baker (Phillipsburg, NJ, USA). Formic acid was obtained from Thermo Fisher Scientific (Waltham, MA, USA). The chlorogenic acid and rutin used as standards were purchased from Sigma-Aldrich (St. Louis, MO, USA).

### 3.2. Animals

Male ICR mice (aged 5–6 weeks, weighing 18–20 g) were obtained from Samtako (Osan-si, Gyeonggi-do, Republic of Korea). The animal room environment was maintained at a temperature of 22 °C ± 3 °C, relative humidity of 50% ± 20%, ventilation of 10–15 air changes/h, and a light/dark cycle of 12 h. The animals were given ad libitum access to food and water. The Institutional Animal Care and Use Committee (IACUC) of the Jeollanamdo Institute for Natural Resources Research approved this study (approval no. JINR1403). All animal experiments were conducted according to the IACUC guidelines. The mice were randomly divided into five groups: the normal group, control group, positive group, LV water extract group, and LV methanol extract group.

### 3.3. Analysis of Fecal Pellet Output Restraint Stress- or 5-HT-Induced Diarrhea in Mice

ICR mice were subjected to restraint stress by individually placing them in 50 mL Falcon tubes for 3 h. These tubes were sufficiently small to restrain the mouse so that it could breathe but not move freely. Mice in the normal group were allowed to move freely inside their cages [28]. The number of fecal pellets excreted during 1 h of restraint stress was measured. Before subjecting the animals to restraint stress, the LV water and methanol extracts were administered at 300 mg/kg for 2 h. Positive control animals were administered PBS.

In the second experiment, mice were administered one of selected different drugs that stimulate intestinal motility, resulting in diarrhea. 5-HT (3 mg/kg) was administered intraperitoneally and subcutaneously. The LV extract was dissolved in PBS and orally administered for 2 h before the animals were subjected to 5-HT treatment. Positive control animals were administered romosertron (30 µg/kg, p.o.).

### 3.4. Expression of the Human 5-HT_3_ Receptor Gene

Human 5-HT_3_ receptor complementary DNA (cDNA) (NM_000869) was purchased from OriGene Technologies (Rockville, MD, USA). The 5-HT_3_ receptor gene was subcloned into the mammalian expression vector pcDNA3.1(+). Chinese Hamster Ovary (CHO)-K1 cells were purchased from the American Type Culture Collection. They were cultured in RPMI 1640 supplemented with 10% FBS at 37 °C in a humidified atmosphere containing 5% CO_2_. To measure [Ca^2+^]*_i_*, CHO-K1 cells were seeded into a 96-well black wall/clear bottom plate (BD Falcon, Franklin Lakes, NJ, USA) at approximately 1 × 10^5^ cells/mL and incubated overnight. Cells were transiently transfected with the human 5-HT_3_ receptor plasmid for 48 h using Lipofectamine^TM^ 2000 (Invitrogen, Carlsbad, CA, USA) according to the manufacturer’s instructions.

### 3.5. Measurement of Intracellular Ca^2+^ ([Ca^2+^]_i_)

To measure 5-HT_3_ receptor-related activity, CHO-K1 cells transfected with the human 5-HT_3_ receptor in a 96-well black wall/clear bottom plate were washed once with N-(2-hydroxyethyl)piperazine-N′-2-ethanesulfonic acid (HEPES)-buffered solution supplemented with 150 mM NaCl, 5 mM KCl, 1 mM MgCl_2_, 2 mM CaCl_2_, 10 mM HEPES, and 10 mM glucose; the pH of the solution was adjusted to 7.4 with NaOH. The cells were loaded with 5 μM fura-2-acetoxymethyl ester (Molecular Probes, Eugene, OR, USA) and 0.001% pluronic F-127 (both fluorescent Ca^2+^ indicators) at 37 °C in a 5% CO_2_ incubator. After incubation for 60 min, the cells were washed three times with HEPES-buffered solution and maintained at a volume of 80 µL/well in the 96-well plates. Cells were monitored at an excitation wavelength of 340/380 nm. For antagonist experiments, cells were pre-incubated with the LV extract for 1 min before adding a 5-HT agonist. All data were collected and analyzed using the BD Pathway 855 system and AttoVision imaging software (v. 1.6) (BD Biosciences, San Jose, CA, USA).

### 3.6. Preparation of the Extract and Standard Solutions

Approximately 50 g of the crushed aerial parts of LV were extracted with 1000 mL of water or methanol in a Soxhlet apparatus for 3 h at 100 °C or 60 °C. The extracts were collected, followed by the addition of 1000 mL of water or methanol to the residue. The extraction process was repeated once. After filtration, the extract was concentrated in a rotary evaporator at 40 °C under decreased pressure and then freeze-dried. The extracted powder was stored at 4 °C before use. Methanolic extract powder was dissolved in methanol at a concentration of 1.0 mg/mL. The sample solution was then filtered using a 0.22-μm syringe filter prior to injection into the HPLC system. Two standard stock solutions (chlorogenic acid and rutin) were prepared at a concentration of 1 mg/mL using HPLC-grade methanol. The standard solutions used in the validation procedure were obtained by diluting the stock solutions with methanol, obtaining final concentrations of 2.5, 5, 10, 25, 50, and 100 μg/mL for each compound.

### 3.7. HPLC Instrumentation and Analytical Method

HPLC analysis was performed on the Waters Alliance e2695 liquid chromatography system (Waters, Milford, MA, USA) equipped with a 2998 PDA detector (Waters). A reverse-phase YMC-Pack Pro C18 column (4.6 mm × 250 mm, 5 μm) was used to perform chromatographic separation at 25 °C. The mobile phase comprised methanol as solvent A and 0.1% formic acid aqueous solution as solvent B. Gradient elution was performed as follows: a linear gradient of 10–20% (A) over 0–5 min, 20–40% (A) over 5–10 min, 40–60% (A) over 10–5 min, and 60–80% (A) for 15–20 min, then returned to 10% (A) (20–20.10 min) and isocratic elution with 10% (A) over 20.10–25 min. The flow rate was maintained at 1.0 mL/min, and the injection volume was 20 μL. The detection wavelength was 324 nm. Empower 3 software (Waters, Milford, MA, USA) was used to acquire and process the data.

### 3.8. Identification of the Marker Compounds

UPLC–MS analysis was performed using the Waters ACQUITY UPLC (Waters, Milford, MA, USA) system equipped with a PDA eλ detector, and a SYNAPT G2-Si Q-TOF MS. ACQUITY UPLC BEH C18 column (2.1 mm × 100 mm, 1.7 μm) was used to perform chromatographic separation at 30 °C. All the instruments and devices were purchased from Waters Corporation. The mobile phase was comprised of acetonitrile as solvent A and 0.1% formic acid aqueous solution as solvent B. Gradient elution was performed as follows: a linear gradient of 10–25% (A) over 0–12 min and 25–100% (A) over 12–12.5 min, returned to 10% (A) over 12.5–13.5 min, and isocratic elution with 10% (A) over 13.5–15 min. The flow rate was maintained at 0.3 mL/min. The injection volume was 5 μL and the detection wavelength was 210–400 nm. MS data were collected at 50–1000 *m*/*z* in electrospray ionization-negative ion mode using the MS^E^ data acquisition method. The source and desolvation gas temperatures were 120 °C and 300 °C, respectively. The capillary and cone voltages were 3 kV and 40 V, respectively. The desolvation and cone gas flow rates were 600 and 50 L/h, respectively. The collision energies were 6 eV for the low-energy scan and 15–40 eV for the high-energy scan. Mass accuracy was calibrated using sodium formate. Leucine-enkephalin ([M − H]^−^ = *m*/*z* 554.2615) was used as the lock mass calibrant. The LC–MS system was operated using MassLynx software (v. 4.1). UNIFI software (v. 1.9.4) was used for chemical identification and matching.

### 3.9. Validation of the HPLC Method

The HPLC analytical method was validated according to the ICH guidelines (2022). The following parameters were evaluated: specificity, linearity, LOD, LOQ, precision, recovery, and solution stability.

#### 3.9.1. Specificity

Specificity was evaluated based on the ability to detect chlorogenic acid and rutin in the LV samples by comparing and overlaying the chromatograms of the standards with those of the samples. The retention times and degrees of separation of the standards and samples were determined to verify their specificity. In addition, blank analysis was performed to verify the absence of interference from other substances.

#### 3.9.2. Linearity, LOD, and LOQ

The linearity was evaluated using the correlation coefficient (r^2^) calculated from the calibration curve of six standard concentrations (2.5, 5, 10, 25, 50, and 100 μg/mL). To perform measurements at each point, experiments were performed in triplicate and a calibration curve was obtained. LOD and LOQ were calculated based on the standard deviation of the response (σ) and the slope of the calibration curve (S). To obtain LOD and LOQ values, the following equations were used: LOD = 3.3σ/S and LOQ = 10σ/S.

#### 3.9.3. Precision

Precision was evaluated using the RSD values obtained using intra- and inter-day experiments. Precision was verified using three different concentrations in the standard linear range, with low (5 μg/mL), medium (25 μg/mL), and high (50 μg/mL) levels of chlorogenic acid and rutin. Intra-day precision was estimated by performing five replicate experiments using standard solutions of concentrations 5, 25, and 50 µg/mL on the same day. Inter-day precision was determined from replicate experiments performed on three consecutive days using the same standard solution.

#### 3.9.4. Recovery

The accuracy of the proposed method was determined by measuring the recovery. Recovery was assessed by spiking the LV extract with three different concentrations constituting low (5 μg/mL), medium (25 μg/mL), and high (50 μg/mL) levels of chlorogenic acid and rutin. Recovery tests were performed in triplicate at each concentration, with the results expressed as the recovery percentage of the spiked standards. Recovery was calculated based on the peak areas of chlorogenic acid and rutin in the spiked and unspiked samples.

#### 3.9.5. Solution Stability

The solution stability of chlorogenic acid and rutin in standard and extracted sample solutions was evaluated by measuring the percentage differences in peak areas under storage conditions of room temperature and 0 °C–4 °C in a refrigerator for time intervals of 0, 12, 24, and 48 h. The stability of the test solution was determined by comparing the responses of aged and fresh solutions.

### 3.10. Statistical Analysis

Data are presented as mean ± standard error of the mean. One-way analysis of variance was used to perform statistical analysis of the data. GraphPad Prism version 5.00 for Windows (GraphPad Software, San Diego, CA, USA) was used to perform data analysis. Duncan’s multiple range test was performed to assess differences between the groups. Statistical significance was set at * *p* < 0.05, ** *p* < 0.01 and *** *p* < 0.001.

## 4. Conclusions

Our study findings suggest that the methanolic extract of LV suppresses fecal pellet output in an IBS-D-mimicking mouse model. Furthermore, the LV extract inhibits the increase in 5-HT_3_ receptor-induced [Ca^2+^]*_i_*, suggesting that LV can function as a 5-HT_3_ receptor antagonist, which is reportedly useful in patients with IBS-D. To the best of our knowledge, this is the first study to reveal that an LV methanol extract may improve IBS-D through a 5-HT_3_ receptor-antagonistic mechanism. Furthermore, the marker compounds chlorogenic acid and rutin were successfully identified in the LV methanol extract using LC–Q-TOF–MS. In addition, a simple and reliable HPLC method was developed to standardize the plant materials and was validated using linearity, LOD, LOQ, intra- and inter-day precision, accuracy, and solution stability. Collectively, our study data can be used in the medical industry as well as to evaluate the application of LV as a valuable medicinal plant resource. Nevertheless, in the future the safety and efficacy of LV should be assessed in patients with IBS-D in clinical settings in order to develop medicines.

## Figures and Tables

**Table 1:** Calibration curve parameters for chlorogenic acid and rutin in the developed HPLC method.

Compound	Linear Equation	Correlation Coefficient (r^2^)	LOD (μg/mL)	LOQ (μg/mL)
Chlorogenic acid	y = 47,062x − 39,362	0.99969	0.39	1.17
Rutin	y = 20,210x − 1351	0.99999	0.27	0.82

**Table 2:** Intra- and inter-day precision values of chlorogenic acid and rutin using the developed HPLC method.

Compound	Intra-Day Precision (*n* = 5)			Inter-Day Precision (*n* = 3)	
	Actual Conc. of Analyte (μg/mL)	Observed Conc. of Analyte (μg/mL)	RSD (%)	Observed Conc. of Analyte (μg/mL)	RSD (%)
Chlorogenic acid	5.00	5.21	0.07	5.39	0.62
	25.00	23.75	0.39	24.36	0.45
	50.00	48.07	0.22	49.42	0.75
Rutin	5.00	5.05	0.32	5.06	0.32
	25.00	24.69	0.15	24.77	0.24
	50.00	49.68	0.15	49.76	0.30

**Table 3:** Recovery values of chlorogenic acid and rutin using the developed HPLC method (*n* = 3).

Compound	Spiked Conc. (μg/mL)	Observed Conc. (μg/mL)	Recovery (%)	RSD (%)
Chlorogenic acid	5.00	5.22	104.47 ± 1.24	1.18
	25.00	25.41	101.65 ± 0.36	0.36
	50.00	50.81	101.61 ± 0.55	0.54
Rutin	5.00	5.18	103.53 ± 1.75	1.69
	25.00	25.04	100.15 ± 0.50	0.50
	50.00	51.39	102.78 ± 0.54	0.53

**Table 4:** Stabilities of chlorogenic acid and rutin in their solutions (standards and extracted sample) under the storage conditions of room temperature and 0–4 °C observed for different time intervals (12, 24, and 48 h).

Time	% Differences in Peak Areas under Storage Condition of Room Temperature				% Differences in Peak Areas under Storage Condition of 0–4 °C			
	Chlorogenic Acid		Rutin		Chlorogenic Acid		Rutin	
	Standard	Extract	Standard	Extract	Standard	Extract	Standard	Extract
12	1.34	0.13	0.39	0.08	1.18	0.21	0.33	0.04
24	0.83	0.19	0.27	0.22	0.38	0.06	0.02	0.06
48	0.57	0.61	0.59	0.15	0.74	0.25	0.52	0.1

The percentage differences were obtained by dividing the absolute value of the differences between the peak areas of the marker compounds in the standard solutions and the LV extract (after 12, 24, and 48 h) and at 0 h by the peak area of those compounds at 0 h (*n* = 3). The results were multiplied by 100 and converted to percentages.

**Table 5:** Contents of chlorogenic acid and rutin in LV methanol extract (*n* = 3).

Compound	Content (mg/g)	RSD (%)
Chlorogenic acid	24.31 ± 0.23	0.95
Rutin	54.89 ± 0.38	0.69

## Data Availability

The data presented in this study are available in article.
